# Application of first-order feature analysis of DWI-ADC in rare malignant mesenchymal tumours of the maxillofacial region

**DOI:** 10.1186/s12903-021-01835-2

**Published:** 2021-09-23

**Authors:** Baoting Yu, Chencui Huang, Shuo Liu, Tong Li, Yuyao Guan, Xuewei Zheng, Jun Ding

**Affiliations:** 1 Department of Radiology, China-Japan Union Hospital of Jilin University, No. 829 of Xinmin Street, Chaoyang District, Changchun, 130021 China; 2 Department of Research Collaboration, R&D Center, Beijing Deepwise and League of PHD Technology Co. Ltd., Beijing, 100080 China

**Keywords:** Maxillofacial, Mesenchymal tumours, MRI, DWI, Radiomics feature, First-order feature

## Abstract

**Background:**

To research the first-order features of apparent diffusion coefficient (ADC) values on diffusion-weighted magnetic resonance imaging (DWI) in maxillofacial malignant mesenchymal tumours.

**Methods:**

The clinical data of 12 patients with rare malignant mesenchymal tumours of the maxillofacial region (6 cases of sarcoma and 6 cases of lymphoma) treated in the hospital from May 2018 to June 2020 and were confirmed by postoperative pathology were retrospectively analyzed. The patients were all examined by 1.5T magnetic resonance imaging. PyRadiomics were used to extract radiomics imaging first-order features. Group differences in quantitative variables were examined using independent-samples t-tests.

**Results:**

The voxels number of ADC_mean_ and ADC_median_ of sarcoma tissues were 44.9124 and 44.2064, respectively, significantly higher than those in lymphoma tissues (ADC_mean_ (− 68.8379) and ADC_median_ (− 74.0045)), the difference considered statistically significant, so do the ADC_kurt_ and ADC_skew_.

**Conclusions:**

The statistical difference of ADC_mean_ and ADC_median_ is significant, it is consistent with the outcome of the manual measurement of the ADC mean value of the most significant cross-section of twelve cases of lymphoma. Development of tumour volume based on the ADC parameter map of DWI demonstrates that the first-order ADC radiomics features analysis can provide new imaging markers for the differentiation of maxillofacial sarcoma and lymphoma. Therefore, first-order ADC features of ADC_kurt_ combined ADC_skew_ may improve the diagnosis level.

## Background

The rapid development of MRI in the maxillofacial region has improved the recognition and diagnosis of maxillofacial tumours significantly. The vast majority of malignant maxillofacial tumours originate from epithelial tissue, and mesenchymal tumours are rare. Therefore, the imaging manifestations of these diseases are not well understood, and the rate of clinical misdiagnosis is high. Different types of tumours have different clinical treatment methods. Sarcoma is generally entirely removed by surgery. Chemotherapy is the standard lymphoma treatment. Therefore, it is vital to determine the type of tumour before operating. DWI could provide essential biomarkers in several kinds of tumours [1, 2].

Radiomics is a new technology whereby algorithms automatically extract and transform a large amount of representative imaging data into exploitable feature spaces that reflect the microscopic characteristics of tumours [3, 4]. MRI could detect and locate the focus and monitor the disease progression that a biopsy cannot [5, 6]. Recently, radiomics has played an important role in the identification of imaging biomarkers and clinical management [7–11]. However, there are few reports on the application and related literature of maxillofacial mesenchymal tumours. In this paper, several rare cases of mesenchymal tumours were analysed retrospectively to provide more reliable clinical evidence for the differential diagnosis of sarcoma and lymphoma. First-order features radiomics analysis, as an emerging tool in MRI analysis [12, 13], could provide insight into tumour heterogeneity and was valuable for differentiating tumour type [14–19].

In this study, the first-order features of the volume of interests (VOI) were extracted from DWI-ADC parameters. These results, together with the mean ADC were analysed for characterisation of rare malignant mesenchymal tumours in the maxillofacial region.

## Methods

### Patients and MRI acquisition parameters

#### Patients

Twelve patients were retrospectively reviewed (seven men, five women; mean age was 54 [range 13–85] years) with a biopsy-proven malignant mesenchymal tumour. The sample patients had preoperative MRI scans between May 2018 and June 2020, and the pathological differentiation could be determined. The sample included six sarcomas and six lymphomas, including one low-grade central osteosarcoma of the left zygoma, three well-differentiated chondrosarcomas of the jaw, two leiomyosarcoma of neck and jaw, two well-differentiated diffuse large B-cell tumour of the buccal region, one B-cell lymphoma related marginal area of tongue mucosa and three non-Hodgkin's follicular lymphoma of the parotid gland. Only patients with sarcomas showed varying degrees of pain and limited facet joint movement, which had no specific clinical symptoms.

The inclusion criteria: biopsy-proven malignant mesenchymal tumour without the concomitant disease. The exclusion criteria were as follows: without definitive post-operative information on pathological characteristics, a minimum tumour diameter < 5 mm, poor MRI quality.

This study was conducted in accordance with the Helsinki Declaration of 1975, as revised in 2013 and approved by the ethics committee of China-Japan Union Hospital of Jilin University in May 2018. All patients signed an informed consent form for inclusion in the study.

#### MRI acquisition parameters

1.5-T Siemens Avanto with an eight-channel phased-array neck coil was used in this study. The patient's head was secured. Non-contrast axial, sagittal and coronal FS-T2WI sequences acquired in multiple breath-holds were obtained by the following parameters: a repetition/echo time of 5080/87 ms, a slice thickness/interslice gap of 4.0/0.4 mm, 20 slices and a matrix of 256 × 320. Axial T1-weighted images were also acquired in multiple breath-holds. Diffusion-weighted images were obtained in the coronal plane. Following the image acquisition, a pixel-wise ADC map was generated by the inbuilt software using b values of 800 s/mm^2^. All patients received a 15-ml intravenous bolus injection of gadodiamide (GE Healthcare Ireland Limited, County Cork, Republic of Ireland). The contrast imaging was performed using a fat-suppressed three-dimensional (3D) T1-weighted volumetric interpolated breath-hold examination sequence after the injection.

The shape, size, signal, bone destruction, adjacent tissue relationship on MRI were evaluated. Besides, the ADC map was generated based on DWI, and the sampling was selected to measure the ADC value at the maximum level of the lesion. The lesions were resected surgically in all eight patients. Histopathological and immunohistochemical staining (IHC) was performed postoperatively.

### MRI and radiomics analysis

**Fig. 1:**
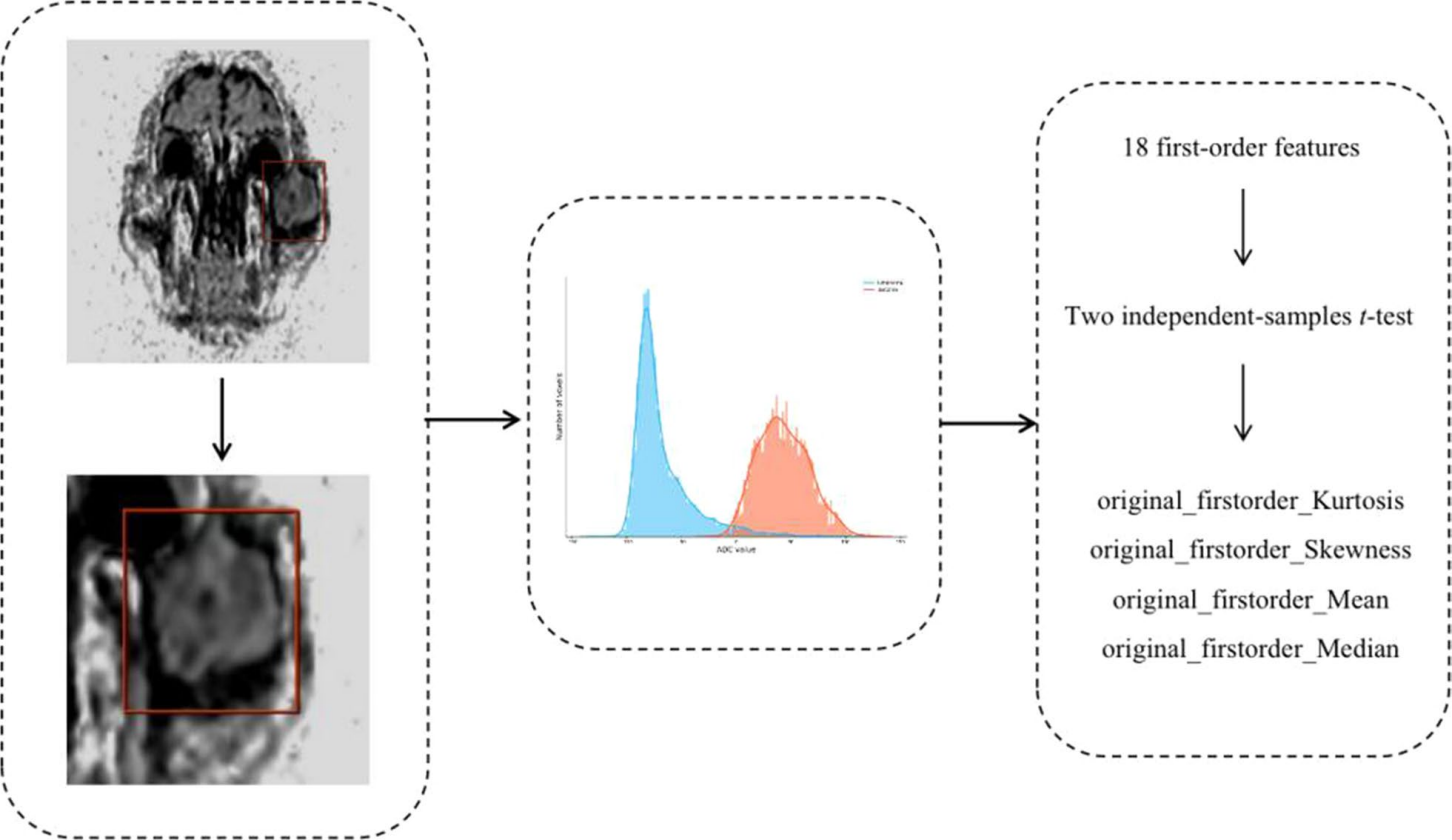
First-order ADC radiomics analysis scheme used in this study

Dr Wise Multimodal Research Platform was used for radiomics analysis. An open-source python package called PyRadiomics (2.2.0) was used for extraction of features. The platform supports feature extraction used to calculate single values per feature for a region of interest (ROI) (‘segment-based’) or generate feature maps (‘voxel-based’) (Fig. 1).

### Delineation of tumour ROI

The tumour regions in the primary dataset were labeled manually by two experts. In the case of disagreement, a third opinion was requested. The DWI-ADC parameter diagram scan was selected as the labeling image, then tumour tissue was classified.

### Extracting features from MRI scans

A B-spline interpolation resampling was used and the anisotropic voxels were resampled to form isotropic voxels of 2.0 mm × 2.0 mm × 2.0 mm. The MRI images were then normalised by centring it at the mean with standard deviation.

$$f(x) = \frac{s(x - \mu x)}{{\sigma x}}$$

(s = 100; μ_χ_ represents mean value; σ represents standard deviation).

Eighteen first-order features were obtained from the original images based on the pixel value extracted from each ROI: Energy, Total Energy, Mean Absolute Deviation, Robust Mean Absolute Deviation, Entropy, 10Percentile, 90Percentile, Minimum, Maximum, Mean, Median, Interquartile Range, Range, Root Mean Squared, Skewness, Kurtosis, Variance and Uniformity.

### Statistical analysis

The research data was normally distributed with homogeneity of variance. All data analyses were carried out using SPSS 16.0 (IBM Corp., Armonk, NY, USA). Group differences in quantitative variables were compared using student’s *t-*test. A *P*-value < 0.05 was considered statistically significant.

## Results

### Imaging and radiomics features

The general characteristics of the twelve study participants were listed in Table 1. The sarcomas showed slightly higher signal intensity with b = 800 s/mm^2^, while ADC images showed a slightly lower signal intensity on DWI images. The mean ADC for all patients with sarcoma was approximately 1.40 × 10^−3^mm^2^/s, while the mean ADC for patients with lymphoma was approximately 0.49 × 10^−3^mm^2^/s. A total of 18 first-order features were obtained. ADC first-order radiomics features were as follows: The mean voxels number of ADC_kurt_ and ADC_skew_ values of sarcomas were 4.1834 and 0.4956 respectively, the values of lymphomas were 9.6219 and 1.8514 respectively. The ADC_kurt_ and ADC_skew_ differed significantly between sarcoma and lymphoma (P < 0.05). The voxels number of ADC_mean_ and ADC_median_ of sarcomas were 44.9124 and 44.2064 respectively, which is significantly higher than that of lymphoma (ADC_mean_ = − 68.8379 and ADC_median_ = − 74.0045), the difference was statistically significant, shown in Table 2.

**Table 1:** The clinical and MRI characteristics of patients (n = 12)

MR1 scan	Sex	Age	Region	ADC valuc (l0 ^3^ mm^2^/s)	Pathological and IHC
Patient 1	F	26	Zygoma	1.12	spindie shaped tumor cells and scattered in trabecular bone tissue and bone like matrix tissue; Ki-67 (10–20%), CK (−), SMA and CD99 (+)
Patient 2	F	40	Jaw	1.56	a large number of chondrocytes with obvious heteromorphism and bone septum; viaentin (+), S-100 and CK (−), Ki-67(60%)
Patient 3	M	56	Jaw	1.54	*A* large number of chondrocytes; S-100(−), Viacntin(+), CK(−), Ki-67(50%)
Patient 4	M	85	Neck	1.25	*A* large number of spindle cells arranged in bundles or vortices, and the cells were slightly deformed, with visible nuclei and mitotic images; viaentin (+), S-100 and CK (−), Ki-67(30%)
Patient *5*	F	62	Jaw	1.36	A large number of spindle cells; Ki-67(10–20%), CK(−), SMA (+)
Patient 6	F	59	Jaw	1.55	a large number of chondrocytes with obvious heteromorphism and bone septum; viaentin (+), Ki-67(30%)
Patient 7	F	62	Buccal	0.56	Lymphoid hyperplasia; PCK(−), EMA(−), CD20(+), CD79a(+), PAX-5(+), CD3(−), CD38(+), CyclinDI(−), MUMl(−), CD30(−), CD 10(+), Bcl-6(−), Bcl-2(−), CD23(−), CD5(+), Ki67(70%)
Patient *8*	M	85	Tongue	0.31	Lymphoid hyperplasia, destroyed lymphoid follicles structure; CD3T(+), BCL-6(−), BCL-2(+), CD 10(−), cyclinDl(−), CD79a(+), Pan-5(+), kappa(−), Ki-67(< 10%)
Patient 9	M	56	Parotid gland	0.37	Lymphoid hyperplasia with obvious heteromorphism; CD3(+), CD20(+), BCL-2(+), BCL-6(+), CD 10(+), Muml(+), PAX-5(+), CD79a(+), Ki-67(70 -80%)
Patient 10	M	67	Parotid gland	0.54	Lymphoid hyperplasia, tumor cells infiltrated glands in some areas, serous acini and adipose tissue display; CD20(+), CD 10 (+), CD3 partial cells (+), CD21 showed FDC network, BCL-6 (+), BCL-2 (+), CD38 germinal center positive (+)
Patient 11	M	13	Parotid gland	0.57	Lymphoid hyperplasia, CD3T(+), CD10(−), BCL-6(+), BCL-2(+), CD79a(+), Ki-67(40–50%)
Patient 12	M	40	Buccal	0.61	Lymphoid hyperplasia, CD3T(+), CD20(−), CD10(−), BCL-6(+), BCL-2(+), CD3 partial cells (+), Ki-67(20%)

**Table 2:** Results of the two-sample t-test (ADC_kurt_, ADC_skew,_ ADC_mean_ and ADC_median_)

First-order features	ADC_kurt_	ADC_skew_	ADC_mean_	ADC_median_
P-value	0.02	0.02	0.01	0.01

A P-value < 0.05 was considered statistically significant

**Fig. 2:**
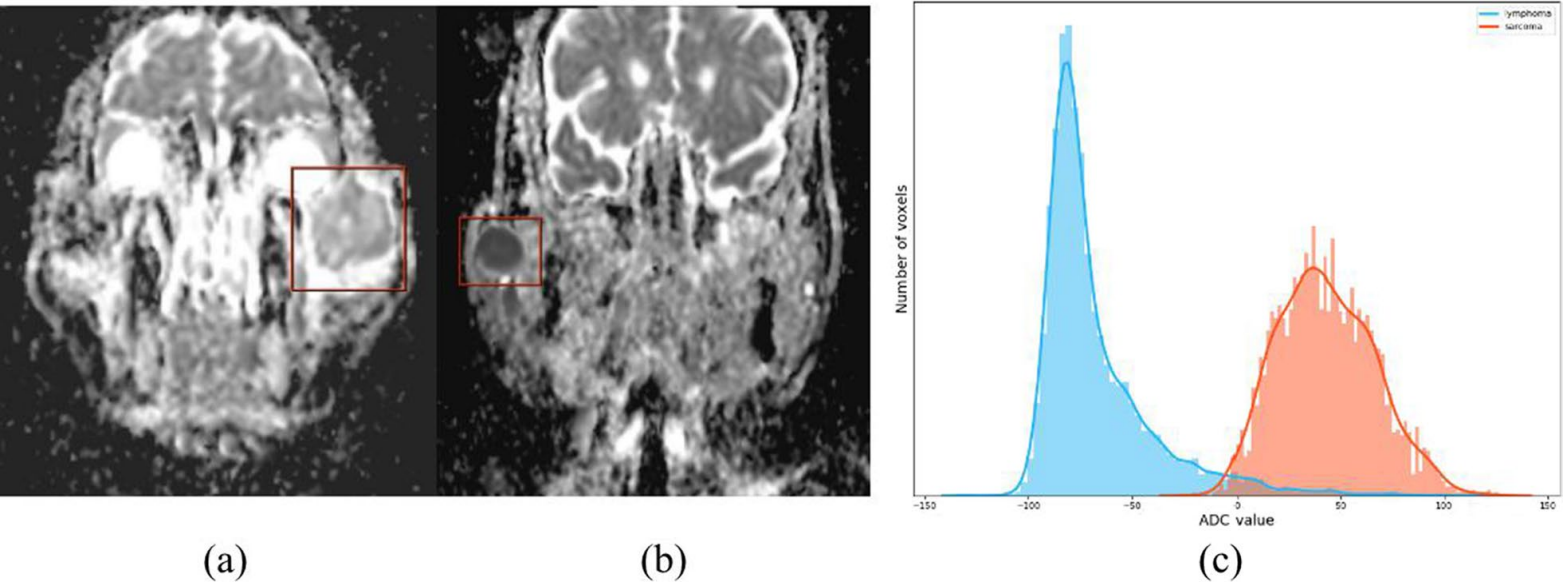
DWI-ADC images of **a** scarcoma and **b** lymphoma. **a** A 28 years old female patient with a sarcoma within the left zygoma, DWI-ADC image the lesion appears relatively inhomogeneous slightly higher signal compared to the adjacent muscle. ADC value is about 1.12 mm^2^/s; **b** a 67 years old male patient with a lymphoma within the right parotid gland, DWI-ADC image the lesion appears relatively homogeneous lower signal compared to the adjacent muscle. ADC value is about 0.54 mm^2^/s; **c** lymphoma correlating with an ADC_mean_ in the lower range, positive skew (high skewness), and a steep curve (high kurtosis). Sarcoma correlating with a higher ADC_mean_ value, a negative skew (low skewness), and a flatter shape (low kurtosis)

Figure 2a shows a low-grade central osteosarcoma of left zygoma with slightly higher ADC value; Fig. 2b shows a non-Hodgkin's follicular lymphoma of the right parotid gland with low value. Figure 2c shows that lymphoma correlates with an ADC_mean_ in the lower range, positive skew (i.e. high skewness) and a steep curve (i.e. high kurtosis). Sarcoma correlates with a higher ADC_mean_ value, a negative skew (i.e. low skewness), and a flatter shape (i.e. low kurtosis).

## Discussion

In this study, the ADC_kurt_, and ADC_skew_ were significant predictive factors. The ADC_mean_ and ADC_median_ were significantly different with statistical significance, the result is consistent with the mean of ADC value. The average ADC value of sarcomas is approximately 1.40 × 10^–3^ mm^2^/s, and the average ADC value of lymphomas is approximately 0.49 × 10^–3^ mm^2^/s. Maeda et al. [20] and Wang et al. [21] found that the average ADC value of lymphoma was lower than head and neck malignant tumours. Furthermore, Sumi et al. [22] reported that an ADC smaller than 0.560 × 10^−3^ mm^2^/s could differentiate pharyngeal lymphomas from pharyngeal carcinomas. These results are consistent with our study. Also, the mean voxels number of ADC_mean_ and ADC_median_ of sarcomas were 44.9124 and 44.2064, respectively, which is significantly higher than that of lymphoma (ADC_mean_ = − 68.8379 and ADC_median_ = − 74.0045), the difference was statistically significant. These results are consistent with the consequences of manual measurement of the maximum cross-section ADC of twelve cases. However, the previous study is limited to the review of ADC; there is no distinction between single lymphoma and sarcoma. The cases in this study are rare, which may enrich the clinical data and further improve the understanding of the radiomics features of mesenchymal tumours.

This study found that ADC_kurt_ and ADC_skew_ differed significantly between sarcoma and lymphoma. Lymphoma correlated with an ADC_mean_ in the lower range. Sarcoma correlated with a higher ADC_mean_ value. This study demonstrates that first-order ADC radiomics analysis, used with the average ADC value to improve diagnostic accuracy, could provide new imaging markers for the differentiation of maxillofacial sarcoma and lymphoma. However, more cases need to be studied. Furthermore, there is theoretical support for studying the difference between the first-order characteristics of sarcoma and lymphoma. Besides, Lisson et al. [23] identified several first-order parameters in order to differentiate enchondroma and low-grade osteosarcoma. Meyer et al. [24] reported that texture analysis parameters derived from MRIs could reflect the Ki67 index in soft tissue sarcoma. So, radiomics analysis can reflect microstructure differences between these tumour entities.

DWI has been used in head and neck tumor imaging and is considered as a sensitive marker for monitoring treatment response in head and neck cancer [25]. While many researchers focused on the interpretation of conventional CT and MRI, the MRI's imaging extraction feature presents an intriguing way to differentiate sarcoma and lymphoma [26, 27]. Suo et al. [28] reported that ADC_skew_, ADC_kurt_ and ADC_mean_ differed between benign bladder lesions and bladder carcinoma. Wang et al. [29] applied first-order ADC texture analysis in order to differentiate lymphoma from metastatic nodes in the head and neck region.

DWI could detect non-invasively the diffusion of water molecules in living tissues [30]. The characteristics of the dense distribution of lymphoma cells and small extracellular space can decrease the ADC value [22]. However, cystic degeneration and necrosis are common in sarcomas, and ADC value is slightly higher [31]. Although ADC value is helpful for clinical identification of maxillofacial lymphoma and other diseases, the rate of clinical misdiagnosis is high because of a low incidence rate and typical clinical manifestations. Therefore, more diagnostic criteria are needed, thus increasing the accuracy of clinical diagnosis. Conventional MRI examinations mainly reflect the shape, composition and water molecule diffusion of a tumour. Texture analysis can extract and quantify the grey level, roughness and homogeneity of lesions that cannot be distinguished by the naked eye [32]. That allows the practitioner to reflect on the characteristics of lesions more comprehensively and carefully [33]. Recent studies found that histogram-based parameters reflected the different histopathological features in several tumour entities [13, 34]. This relationship maybe help to better characterise tumours through radiological imaging and aid in differentiating between tumour types [35].

Based on the number of cases and features selected in this paper, 18 kinds of first-order features of lesions were extracted for analysis. First-order statistics describe the distribution of voxel intensities. Skewness measures the asymmetry of the distribution of values. A higher kurtosis reflected that distribution mass is concentrated towards the tail(s) rather than towards the mean. Lower kurtosis implies the reverse. Fine texture usually appears in healthy tissue, while rough texture highlights the heterogeneity of the tumour. In this study, the first-order feature analysis of rare cases showed that sarcoma and lymphoma were different in malignant mesenchymal tumours of the maxillofacial region. Therefore, radiomics analysis can provide quantitative parameters in the tumour ROI.

### Limitations

First, the number of patients was limited. Analysing a more significant number of patients’ textural parameters may have shown vital information related to tumour characteristics. Second, the differences relating to the imaging parameters and image viewer remain unknown. Future studies of this nature should include a more significant number of cases.

## Conclusions

In conclusion, the development of tumour volume based on the ADC parameter map of DWI first-order ADC radiomics analysis makes it possible to provide new imaging markers for the differentiation of maxillofacial sarcoma and lymphoma. Primarily, the feature parameters ADC_kurt_ and ADC_skew,_ combined with the mean ADC value, effectively improves the diagnostic level. However, due to the low incidence rate and a limited number of cases, this study explored only first-order features that were closely related to malignant mesenchymal tumours. It can be concluded from the results of the study that tumour classification can be predicted based on radiomics features, and further study is recommended.

## Data Availability

All data generated or analyzed during this study are included in this published article.

## Abbreviations

MRI: Magnetic resonance imaging
DWI: Diffusion-weighted imaging
ADC: Apparent diffusion coefficient
VOI: Volume of interests
ROI: Region of interest
IHC: Immunohistochemical
